# The involvement of extracellular ATP in regulating the stunted growth of *Arabidopsis* plants by repeated wounding

**DOI:** 10.1186/s12870-022-03656-z

**Published:** 2022-06-08

**Authors:** Zhenzhen Shi, Hanqi Wang, Yuejing Zhang, Lingyun Jia, Hailong Pang, Hanqing Feng, Xin Wang

**Affiliations:** College of Life Science, Northwest Normal University, Lanzhou, Gansu, 730070 China

**Keywords:** Repeated wounding, Extracellular ATP, Stunted growth

## Abstract

**Background:**

Extracellular ATP (exATP) has been shown to act as a signal molecule for regulating growth, development, and responses of plants to the external environment.

**Results:**

In this study, we investigated the possible involvement of exATP in regulating the stunted growth caused by repeated wounding. The present work showed that the repeated wounding caused the decreases in leaf area, fresh weight, dry weight, and root length of *Arabidopsis* seedlings, while the exATP level was enhanced by the repeated wounding. Repeated application of exogenous ATP had similar effects on the plant growth, as the repeated wounding. Through the comparison of *p2k1-3* mutant (in which T-DNA disrupted the gene coding P2K1, as exATP receptor) and wide type (WT) plants, it was found that the mutation in P2K1 decreased the sensitivity of plant growth to the repeated wounding and exogenous ATP application. Further works showed that the ibuprofen (IBU, an inhibitor of jasmonate biosynthesis) partially rescued the wound-induced growth degradation. In comparison, the P2K1 mutation partly rescued the wound-induced growth degradation, whereas this mutation failed to do so in the wounded seedlings treated with IBU, indicating that the role of exATP in regulating the growth degradation by repeated wounding could be linked to the JA signaling pathway.

**Conclusions:**

In conclusion, these results indicate that exATP could be a regulator for the stunted growth of plants by repeated wounding.

**Supplementary Information:**

The online version contains supplementary material available at 10.1186/s12870-022-03656-z.

## Background

Adenosine 5’-triphosphate (ATP) is important energy currency molecule for living organisms [1]. It is produced by oxidative phosphorylation within intracellular organelles [2]. In the last decades, it has been found that animal, plant, and microbial cells can release ATP from the intracellular spaces into the extracellular matrix [3–5]. Different from the role of the intracellular ATP as energy currency molecule, extracellular ATP (exATP) is considered to be a signaling molecule for regulating the physiological processes of cells [6].

Plant cells can release exATP via the ATP-binding cassette (ABC) transporter, exocytosis, or plasma membrane-localized nucleotide transporter, such as PM-ANT1 [7–9]. Further research found that environmental stimuli, such as wounding, hypertonic stress, cold, and pathogen infection, may change exATP levels of plants [10–14]. Moreover, many studies found that apyrase plays important roles in limiting exATP level by hydrolyzing exATP [15, 16].

From the current works available, Ca^2+^, nitric oxide (NO), and reactive oxygen species (ROS) act as the downstream signalling molecules of exATP [12, 17–19]. In animal cells, it is well known that exATP regulates physiological processes via binding and stimulating the purinoceptors of the plasma membrane, including metabotropic (P2Y) and ionotropic (P2X) receptors [20]. Choi et al. [21] in *Arabidopsis* revealed that the DORN1 (Does not Respond to Nucleotides 1, a lectin receptor kinase-I.9) protein binds exATP with high affinity. In order to keep with the animal P2 (X and Y) receptor nomenclature, the DORN1 is also called as the P2K1.

The physiological roles of exATP in plants are also widely studied. Many works have revealed that exATP functions in regulating the responses of plants to biotic and abiotic stresses, such as pathogen infection [19], high salt, and cold [22, 23]. Otherwise, exATP is also found to have ability to affect the cell viability, growth, development, and gravitropism of plants [15, 24–26].

Wounding, commonly caused by mechanical injury, pathogen infection, or insect infestation, is a continual threat to the survival of plants [27]. Wounding is known to result in marked changes in the gene expression and physiological responses of plants [28–30]. When plants are repeatedly wounded, the wounded plants become stunted, which is characterized by the reduction of leaves and roots in size [31]. This is thought to be an important reason for the dramatic reduction in yield of crops injured by pathogen or herbivory. And, this also provides an attractive issue about the antagonistic relationship between plant growth and the defense against biotic stresses [19, 26, 29, 32].

As introduced above, wounding provides a passive route of ATP releases [15, 33]. Choi et al. [10] reported that approximately 60% of the exATP-induced genes were also induced by wounding, indicating that exATP plays important role in regulating the responses of the plants to wounding. However, the role of exATP in the wounding responses is commonly evaluated in the plants subjected to single wounding [29, 30]. Under the condition of repeated wounding, it is not well understood whether exATP can be involved in the regulation of the stunted growth.

In the present work, our data indicates that exATP could be a regulator for the stunted growth of plants by repeated wounding. We believe that this study will help to further understand the role of exATP in plants and expand the current knowledge on the regulation of plant growth under stressful conditions.

## Results

### Repeated wounding decreased the growth of *Arabidopsis* seedlings

In the present work, the leaves of the 21-day-old wide type (WT) *Arabidopsis* seedlings were subjected to the repeated wounding. The changes of leaf area were continuously monitored as the indicator of the growth, since the measurement of leaf area did not destroy seedlings and disturb the progress of the growth of the seedlings.

**Fig. 1:**
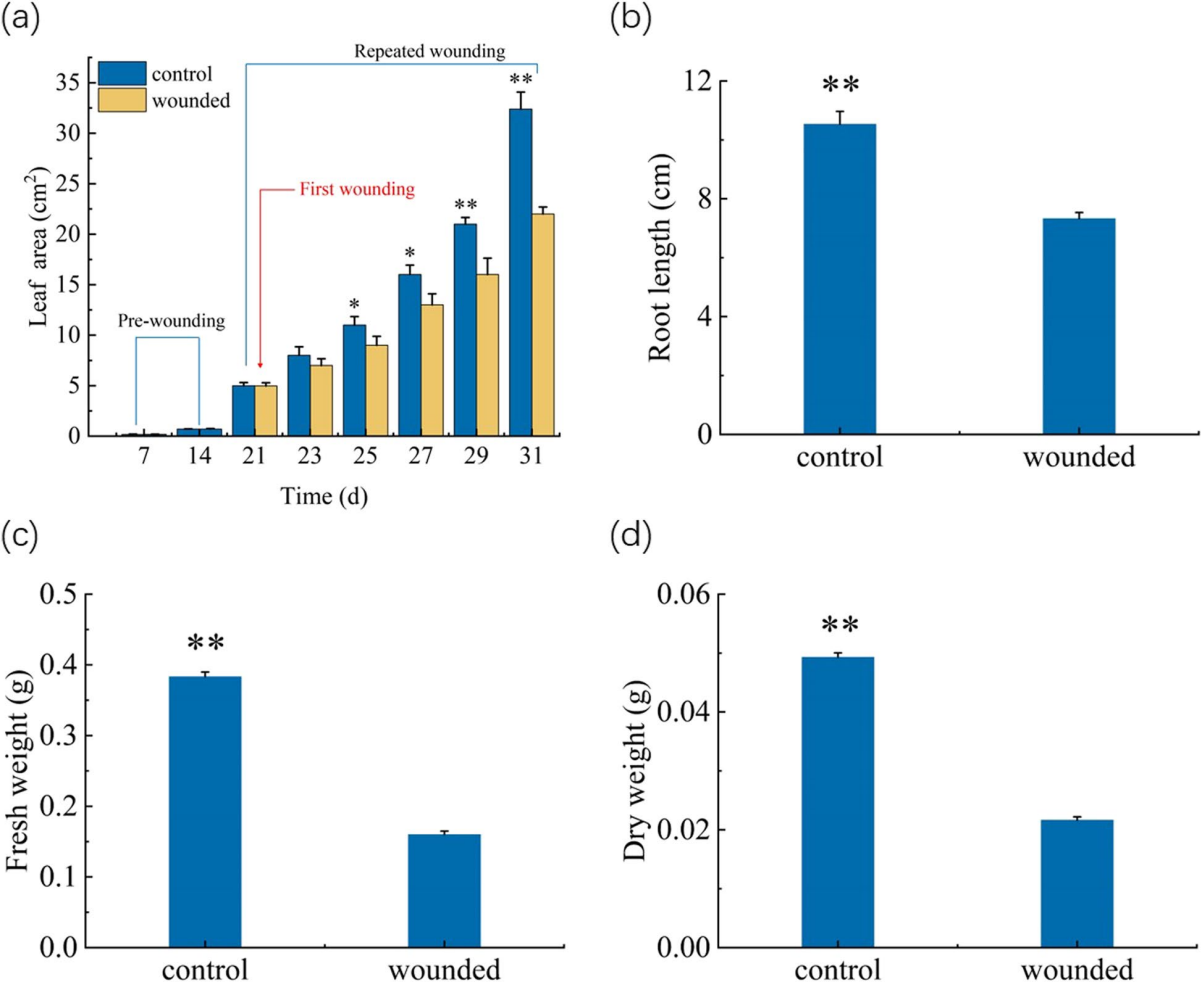
Effect of the repeated wounding on the growth of the WT (wild type) seedlings. **a** Leaf area. **b** Root length. **c** Fresh weight. **d** Dry weight. The first wounding was performed at 21th day of the growth, indicated by the red line. Control: the un-wounded WT seedlings, and wounded: the wounded WT seedlings. The values represent means ± SD from twenty independent seedlings. *—statistically significant differences between the control and wounded treatment (* *P* < 0.05, ** *P* < 0.01)

The results showed that the repeated wounding caused the reduction in leaf area of *Arabidopsis* seedlings (Fig. 1a). We also measured the level of other biomass parameters at 10th day after the first wounding (as the representative time point). It was shown that the levels of fresh weight, dry weight, and root length of the wounded seedlings were significantly less than the un-wounded seedlings (Fig. 1b-d).

### Repeated wounding increased the exATP level of *Arabidopsis* seedlings

**Fig. 2:**
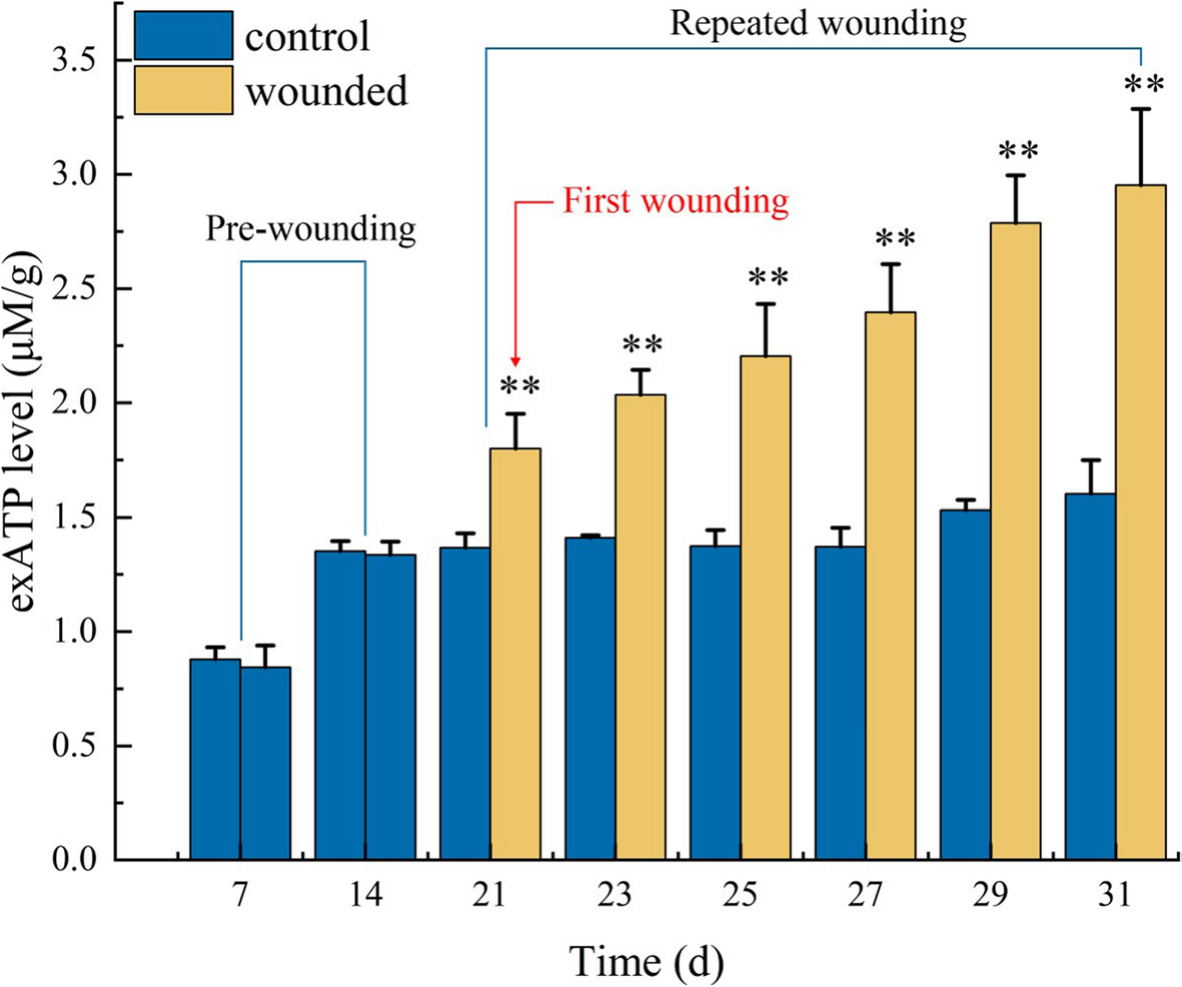
Effect of the repeated wounding on the leaf exATP level of the WT seedlings. The first wounding was performed at 21th day of the growth, indicated by the red line. Control: the un-wounded WT seedlings, and wounded: the wounded WT seedlings. The values represent means ± SD from twenty independent seedlings. *—statistically significant differences between the control and wounded treatment (** *P* < 0.01)

As previously reported, wounding can increase the exATP level of plants [15, 34]. In the present work, the repeated wounding was applied to the leaves of the WT *Arabidopsis* seedlings and the leaf exATP level was assayed. It was observed that the repeated wounding led to continuous increase in the exATP level in the wounded seedlings, compared to the un-wounded seedlings (Fig. 2).

### ExATP play a role in the decreased growth of *Arabidopsis* seedlings by repeated wounding

The results above showed that the repeated wounding decreased the growth but increased exATP level of *Arabidopsis* seedlings (Fig. 2). Thus, we attempted to study whether exATP could play a role in the wound-induced growth degradation.

Because ATP has high charge, ATP applied exogenously cannot freely diffuse across the plasma membrane and thus can increase the exATP level [13]. Hence, in many works, the potential biological functions of exATP can be revealed by exogenous application of ATP [7, 14]. In the present work, the exogenous 0.1–5 mM ATP was applied repeatedly to the WT *Arabidopsis* seedlings, and the effects of exogenous ATP on leaf area was continuously monitored.

**Fig. 3:**
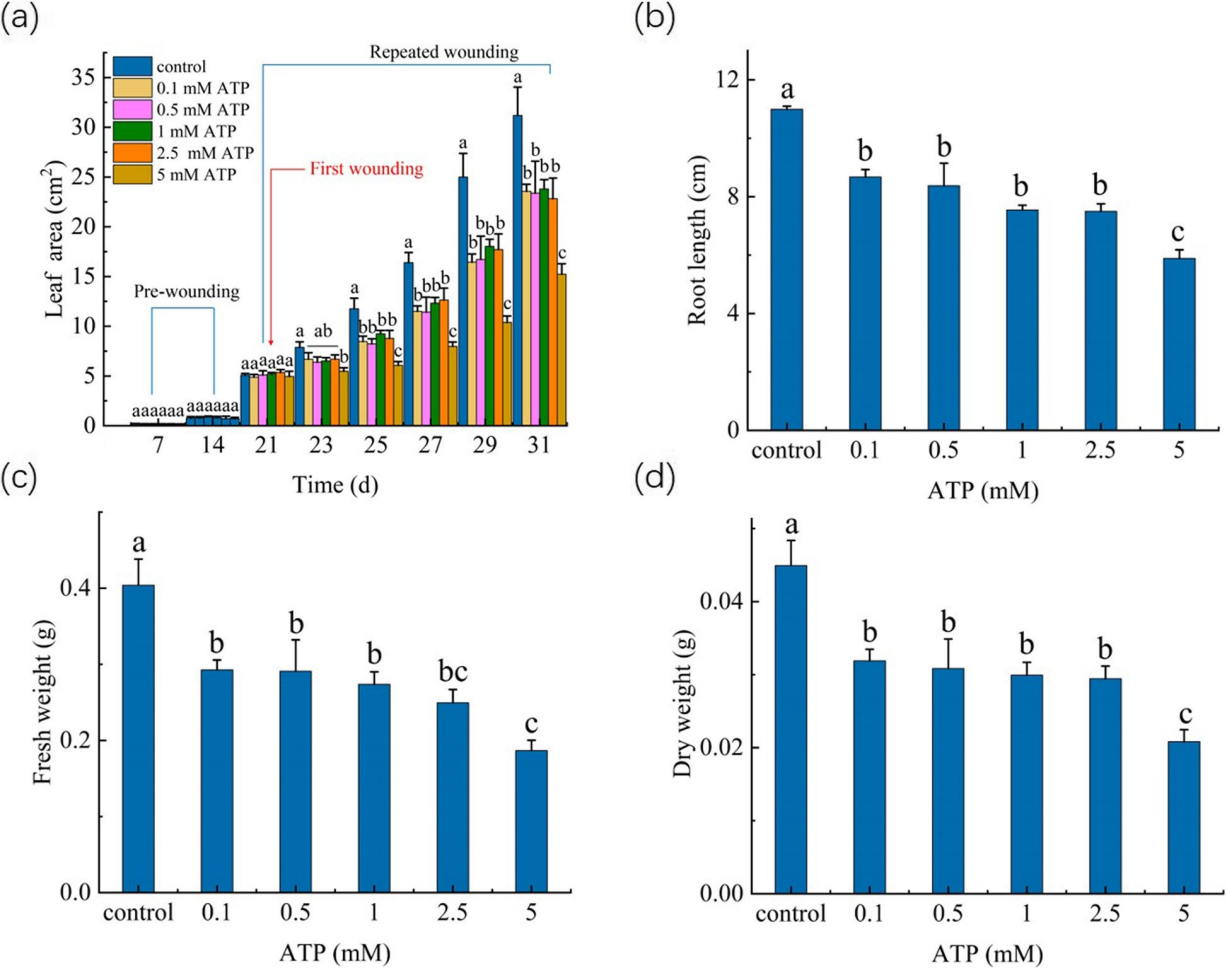
Effect of the repeated application of ATP (0.1—5 mM) on the growth of WT seedlings. **a** Leaf area. **b** Root length. **c** Fresh weight. **d** Dry weight. The first application of ATP was performed at 21th day of the growth, indicated by the red line. Control: the WT seedlings without ATP treatment, and ATP: the WT seedlings repeatedly treated with ATP. The values represent means ± SD from twenty independent seedlings. For Fig. 3a, different letters on top of the bars indicate significant differences (*P* < 0 .05) among the different treatments at the same time. For Fig. 3b-d, the means denoted by the same letter did not significantly differ at *P* < 0.05 among the different treatment

The results showed that the repeated application of exogenous ATP from 0.1–2.5 mM caused the decrease in leaf area of the WT *Arabidopsis* seedlings (Fig. 3a). And, the values of fresh weight, dry weight, and root length at 10th day after the first application of exogenous 0.1–2.5 mM ATP (as the representative time point) were also decreased, compared to those in the WT seedlings without ATP treatment (Fig. 3b-d). Exogenous application of ATP at higher concentration (5 mM) substantially suppressed the growth (Fig. 3). Thus, it seems that the repeated application of exogenous ATP had similar effects on the plant growth, as the repeated wounding.

P2K1, a lectin receptor kinase, has been confirmed to recognize exATP in *Arabidopsis* and is required for the exATP-induced responses to wounding [10]. In the present work, the *p2k1-3* mutant plants, in which T-DNA disrupted the *p2k1* gene at nucleotide 92 of the open reading frame [21], were used to further evaluate the role of exATP in the wound-induced decrease of growth.

**Fig. 4:**
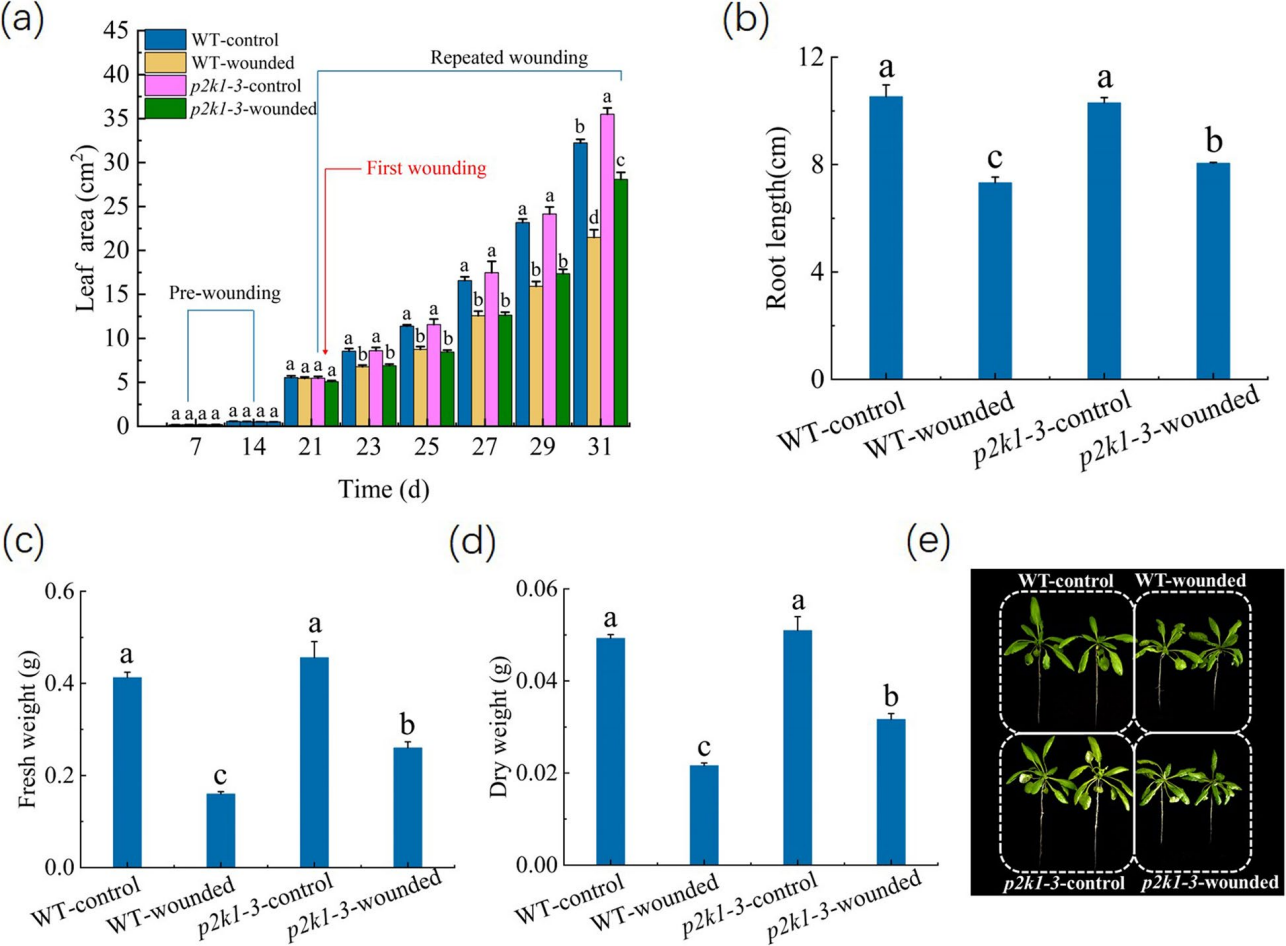
Effect of the repeated wounding on the growth of WT and *p2k1-3* mutant seedlings. **a** Leaf area. **b** Root length. **c** Fresh weight. **d** Dry weight. **e** Photograph of plant growth of WT and *p2k1-3* mutant seedlings was taken at 10th day after the first wounding. The first wounding was performed at 21th day of growth, indicated by the red line. WT-control: the un-wounded WT seedlings, WT-wounded: the wounded WT seedlings, *p2k1-3*-control: the un-wounded *p2k1-3* mutant seedlings, and *p2k1-3*-wounded: the wounded *p2k1-3* mutant seedlings. The values represent means ± SD from twenty independent seedlings. For Fig. 4a, different letters on top of the bars indicate significant differences (*P* < 0 .05) among the different treatments at the same time. For Fig. 4b-d, the means denoted by the same letter did not significantly differ at *P* < 0.05 among the different treatment

Under either the wounded or un-wounded condition, there was no significant difference in the leaf area between the WT and *p2k1-3* mutant plants during the first 29 days of the growth (Fig. 4a). At 31th day of the growth, *p2k1-3* mutant plants displayed larger leaf area than the WT plants under the un-wounded condition (Fig. 4a). We next compared the leaf area of the wounded WT seedlings to the leaf area of the wounded *p2k1-3* mutant seedlings. The results showed that the leaf area of the wounded *p2k1-3* mutant seedlings were also larger than the wounded WT seedlings at 10th day after the first wounding (i.e. at 31th day of growth) (Fig. 4a).

The results above indicate that P2K1 mutation can affect plant growth under either the wounded or un-wounded condition. We further calculated the quantitative difference of the leaf area between the *p2k1-3* mutant and WT seedlings at 31th day of the growth under either the wounded or un-wounded condition. At this time point, the difference of the leaf area between the *p2k1-3* mutant and WT seedlings under the wounded condition (*D *_*leaf-area*_* p2k – wt under wounding*) were 6.60, while the difference of the leaf area between the *p2k1-3* mutant and WT seedlings under the un-wounded condition (*D *_*leaf-area*_* p2k – wt under un-wounding*) was only 3.25 (Table 1). This indicates that the effect of P2K1 mutation on the growth of the wounded plants was more pronounced than that on the growth of the unwounded plants.

**Table 1:** The quantitative difference of the leaf area between the *p2k1-3* mutant and WT plants under wounded (*D *_*leaf-area*_* p2k—wt* under wounding) or un-wounded condition (*D *_*leaf-area*_* p2k—wt* under un-wounding), and the quantitative difference of the leaf area between the unwounded and wounded WT seedlings (*D *_*leaf-area*_* unwounded—wounded* WT) or between the unwounded and wounded *p2k1-3* seedlings (*D *_*leaf-area*_* unwounded—wounded p2k1-3*). The values of the *D *_*leaf-area*_ represent means ± SD from twenty independent seedlings. *- statistically significant differences between the *p2k1-3* mutant and WT plants under wounded or un-wounded condition (* *P* < 0.05, ** *P* < 0.01). ^#^—statistically significant differences between un-wounded and wounded condition in the WT or *p2k1-3* seedlings (^#^ *P* < 0.05, ^##^ *P* < 0.01)

Seedlings ages (days)	*D *_*leaf-area*_* p2k—wt* under wounding	*D *_*leaf-area*_* p2k—wt* under un-wounding	*D *_*leaf-area*_* unwounded—wounded* WT	*D *_*leaf-area*_* unwounded—wounded p2k1-3*
21	-0.37	-0.08	0.09	0.38
23	0.12	0.06	1.78^#^	1.72^#^
25	-0.29	0.19	2.64^##^	3.12^#^
27	0.06	0.90	4.00^#^	4.84^##^
29	1.43	0.97	7.24^##^	6.78^##^
31	6.60**	3.25*	10.75^##^	7.40^##^

Further analysis showed that, at 31th day of growth, the quantitative difference of the leaf area between the unwounded and wounded WT seedlings (*D *_*leaf-area*_* unwounded – wounded WT*) was 10.75, while the difference of the leaf area between the unwounded and wounded *p2k1-3* mutant seedlings (*D *_*leaf-area*_* unwounded – wounded p2k1-3*) was only 7.40 (Table 1). This indicates that the effect of wounding on the growth of the WT plants is more pronounced than that on the growth of the *p2k1-3* mutant plants.

The measurement on fresh weight, dry weight, and root length at 31th day of growth showed that there was no significant difference in these biomass parameters between the WT and *p2k1-3* mutant seedlings under the un-wounded conditions, whereas the wounded *p2k1-3* mutant seedlings had higher levels of fresh weight, dry weight, and root length of than the wounded WT plants at 10th day after the first wounding (Fig. 4b-d). These results also further indicate that the mutation in exATP receptor decreased the sensitivity of plant growth to the repeated wounding.

**Fig. 5:**
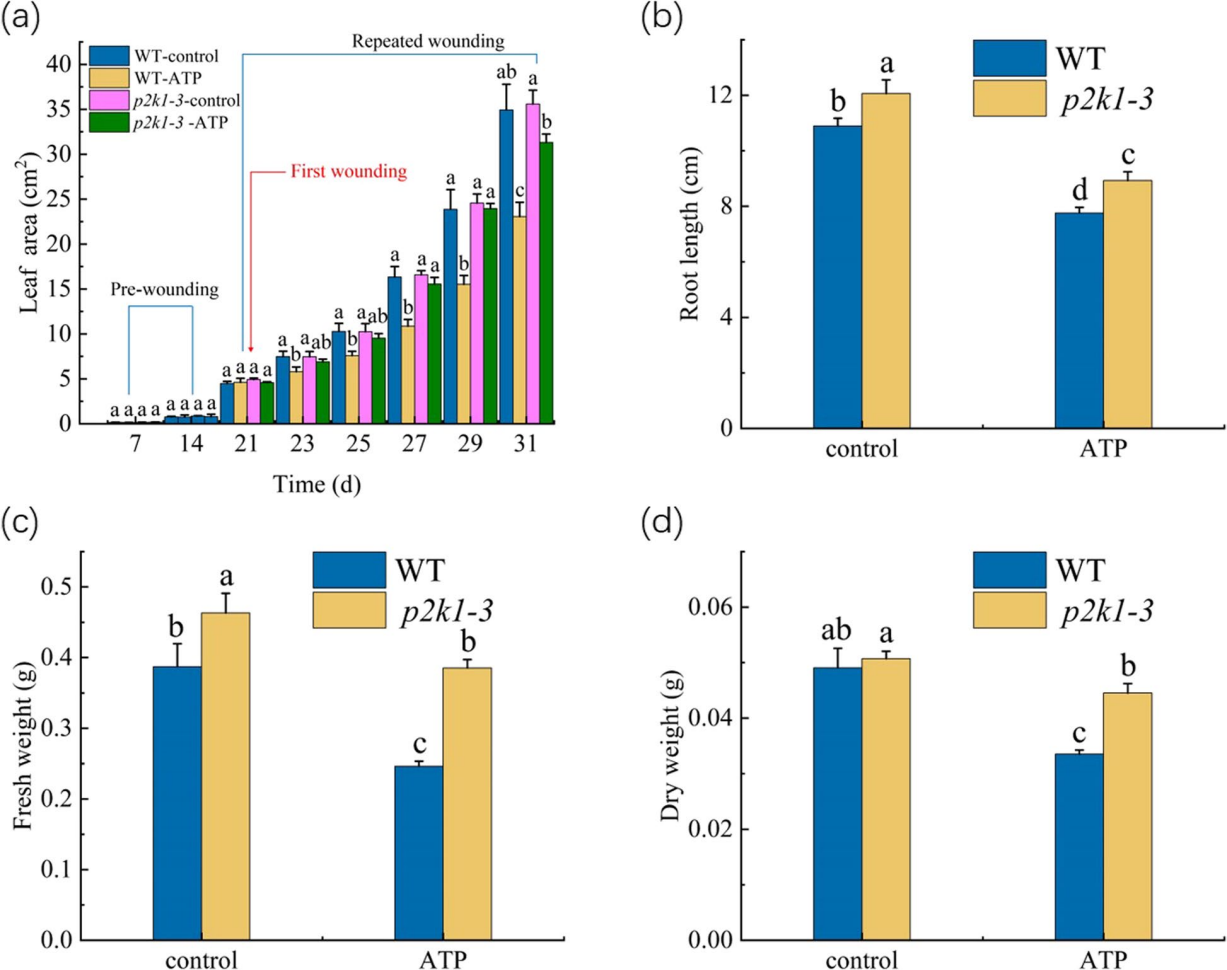
Effect of the repeated application of ATP (1 mM) on the growth of WT and *p2k1-3* mutant seedlings. **a** Leaf area. **b** Root length. **c** Fresh weight. **d** Dry weight. The first application of ATP was performed at 21th day of growth, indicated by the red line. Control: the WT seedlings without ATP treatment, and ATP: the WT seedlings repeatedly treated with ATP. The values represent means ± SD from twenty independent seedlings. For Fig. 5a, different letters on top of the bars indicate significant differences (*P* < 0 .05) among the different treatments at the same time. For Fig. 5b-d, the means denoted by the same letter did not significantly differ at *P* < 0.05 among the different treatment

We also further studied the different effects of exogenous ATP treatment on the growth of the WT and *p2k1-3* mutant *Arabidopsis* seedlings (1 mM ATP was chosen to investigate this issue). Exogenous ATP decreased the leaf area, root length, fresh weight, and dry weight of the either WT or *p2k1-3* mutant *Arabidopsis* seedlings. In comparison, the suppression of the growth of the WT plants by ATP was more obvious than that of *p2k1-3* mutant plants by the same concentration of ATP (Fig. 5). Combined this observation with the results in the Figs. 3 and 4, we suggest that the growth degradation by the repeated wounding is related the exATP.

### Regulation of the wound-induced stunted growth by exATP could be associated with the JA signaling

**Fig. 6:**
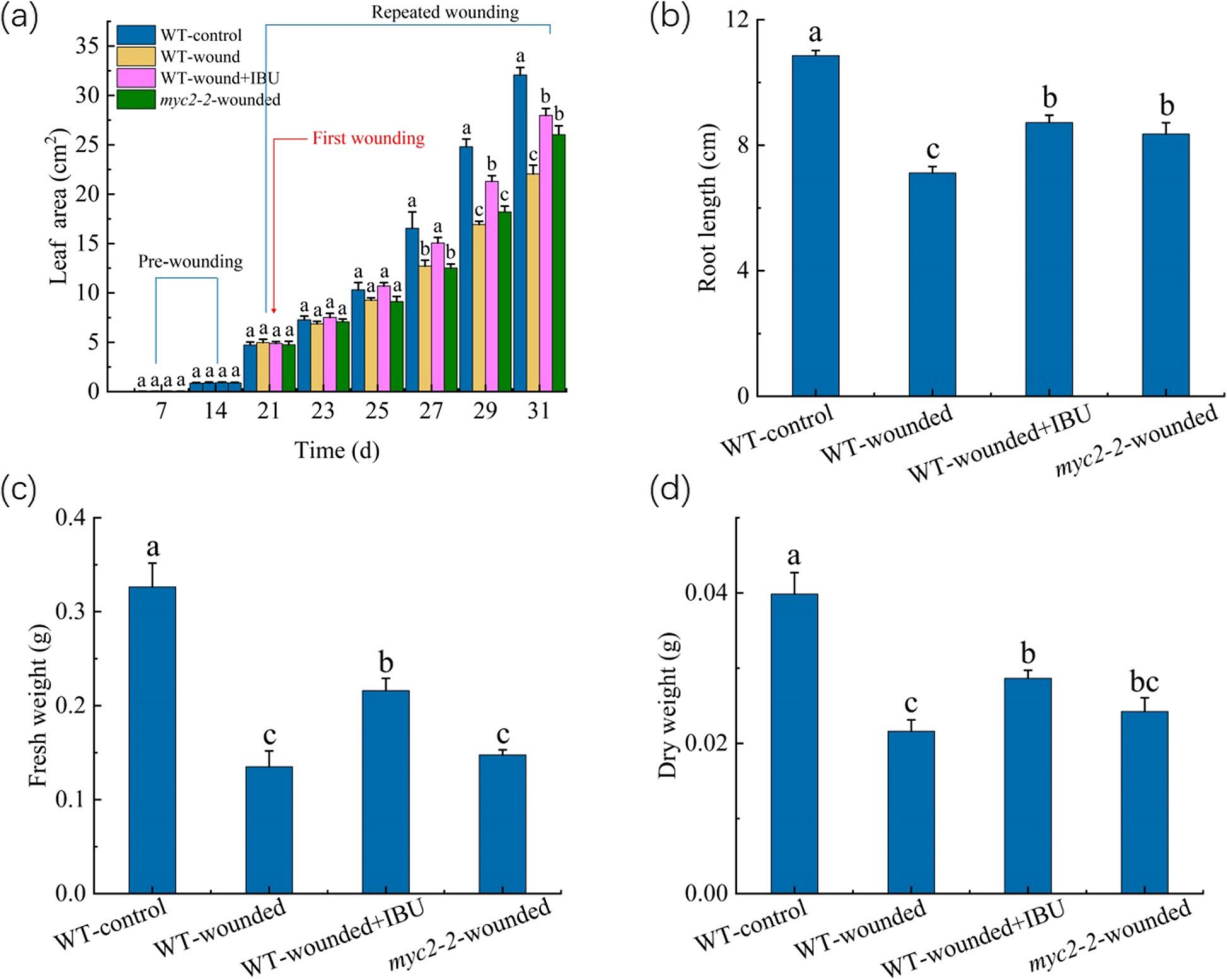
Effect of the repeated wounding on the growth of the seedlings with the IBU application or MYC2 mutation. **a** Leaf area. **b** Root length. **c** Fresh weight. **d** Dry weight. The first wounding, the first IBU application, or first wounding plus IBU application were performed at 21th day of growth, indicated by the red line. WT-control: the un-wounded WT seedlings, WT-wounded: the wounded WT seedlings, WT-wounded plus IBU: the wounded WT seedlings treated with IBU, *myc2-2*-wounded: the wounded *myc2-2* mutant seedlings. The values represent means ± SD from sixteen independent seedlings. For Fig. 6a, different letters on top of the bars indicate significant differences (*P* < 0 .05) among the different treatments at the same time. For Fig. 6b-d, the means denoted by the same letter did not significantly differ at *P* < 0.05 among the different treatment

Previous works showed that JA (jasmonate) level is obviously enhanced by wounding and JA is an important signaling in the responses of plant to wounding [31, 35–38]. Thus, we first evaluated the role of JA in the wound-induced stunted growth by using ibuprofen (inhibitor of JA biosynthesis, IBU) and *myc2-2* mutant plants, in which a master JA-activated transcription factor MYC2 was mutated. Alone application of IBU and mutation of MYC2 under un-wounded condition had no significant effects on the growth of the WT-plants (data not shown). When the plants were subjected to repeated wounding, either IBU application or MYC2 alleviated the decrease of leaf area by the repeated wounding (Fig. 6a). And, further measurement at 10th day after the first wounding (as the representative time point) showed that IBU can partially rescue the wound-induced decrease of growth parameters, including leaf area, root length, fresh weight, and dry weight (Fig. 6b-d). However, MYC mutation only partially rescued the wound-induced decrease of leaf area and root length but had no significant effects on the wound-induced decrease of fresh weight, and dry weight (Fig. 6b-d). These results suggest that the JA-signaling is involved in the regulation of the wound-induced growth degradation, and JA biosynthesis is vital for this process.

We further studied whether the regulation of the wound-induced stunted growth by exATP could be associated with the JA signaling. As the results shown above, either the mutation in exATP receptor or application of IBU partially rescued the wound-induced decrease in growth (Figs. 4 and 6). Thus, in the further work, IBU was applied exogenously to the *p2k1-3* mutation seedlings to evaluate how, or to what extent, the combination of *p2k1-3* mutation and IBU can rescue the wound-induced decrease in growth.

**Fig. 7:**
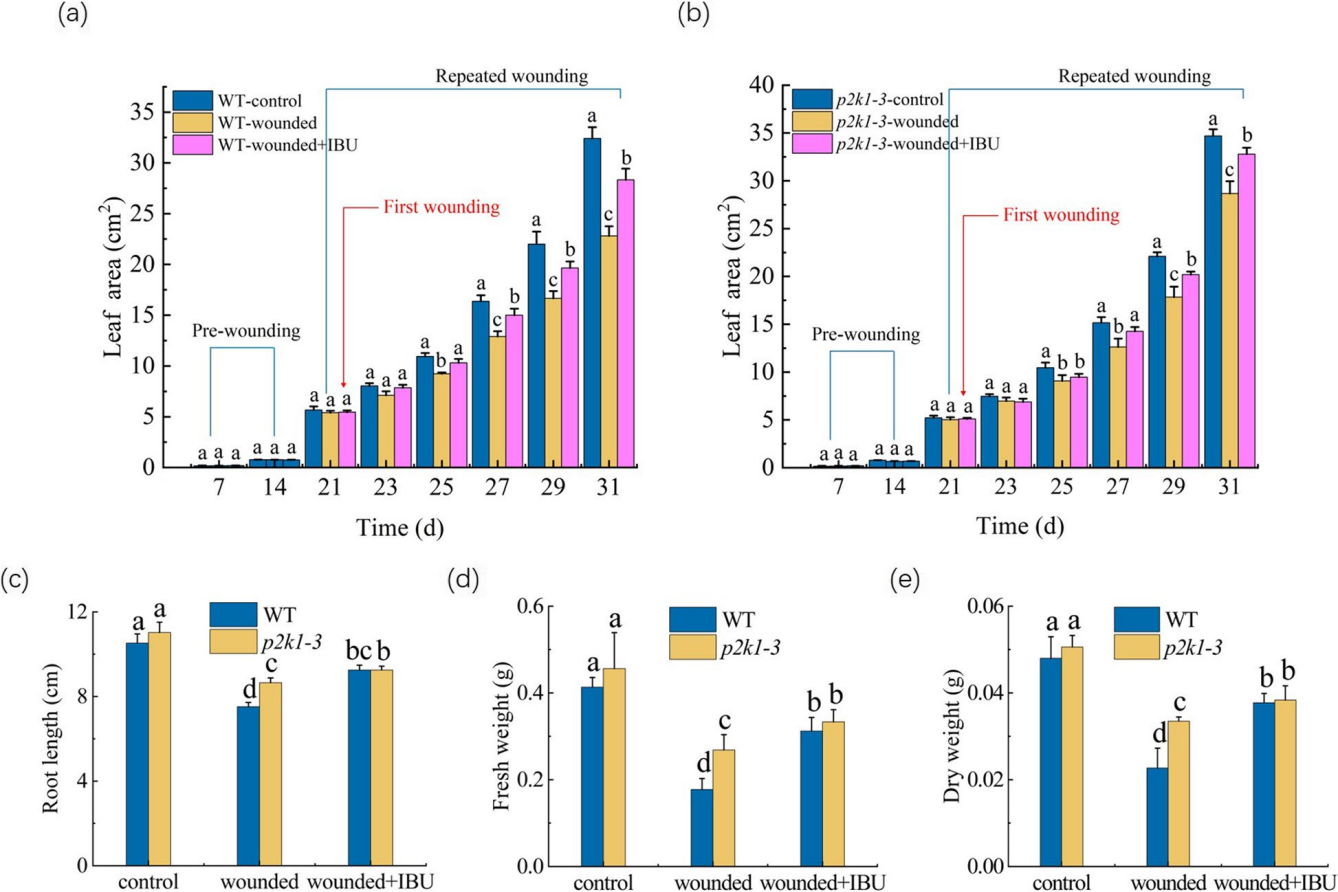
Effect of the repeated wounding on the growth of the seedlings with the IBU application or P2K1 mutation, or the combination of P2K1 mutation and IBU application. **a** Leaf area of WT seedlings. **b** Leaf area of *p2k1-3* mutant seedlings. The first wounding, the first IBU application, or first wounding plus IBU application were performed at 21th day of growth, indicated by the red line. **c** Root length. **d** Fresh weight. **e** Dry weight. WT-control: the un-wounded WT seedlings, WT-wounded: the wounded WT seedlings, WT-wounded plus IBU: the wounded WT seedlings treated with IBU, *p2k1-3*-control: the un-wounded *p2k1-3* mutant seedling, *p2k1-3*-wounded: the wounded *p2k1-3* mutant seedlings, and *p2k1-3*-wounded plus IBU: the wounded *p2k1-3* mutant seedlings treated with IBU. The values represent means ± SD from sixteen independent seedlings. For Fig. 7a-b, different letters on top of the bars indicate significant differences (*P* < 0 .05) among the different treatments at the same time. For Fig. 7c-e, the means denoted by the same letter did not significantly differ at *P* < 0.05 among the different treatment

Alone use of IBU had no significant effects on the leaf area, fresh weight, dry weight, and root length of the *p2k1-3* mutation seedlings under un-wounded condition (data not shown). And, the rescued degree of the wound-induced growth degradation either by *p2k1-3* mutation or that by IBU alone was compared with that by the combination of *p2k1-3* mutation and IBU at 10th day after the first wounding (Fig. 7).

On one hand, it seems from these data that the combination of P2K1 mutation and IBU did not exert an obvious additive rescue efficacy in attenuating the wound-induced growth degradation when compared with either the P2K1 mutation or IBU treatment alone (Fig. 7). On the other hand, the P2K1 mutation partly rescued the wound-induced growth degradation, whereas this mutation failed to do it in the wounded seedlings treated with IBU (Fig. 7). This indicates that the regulation of the wound-induced growth degradation by JA and exATP could be not two independent events.

## Discussion

Plants are constantly exposed to mechanical wounding caused by weather conditions such as wind and by biotic factors, including pathogens and herbivorous animals [29, 39]. The effects of temporary wounding on plants include marked changes in the gene expression and physiological responses, such as the increase of ROS [10, 17, 35, 40]. However, when plants were repeatedly injured, growth of plants is stunted and the size of plant organs such as leaves is greatly reduced [31, 41]. This is the one of the important reasons for the reduced growth and decreased production of crops by herbivores infestation [42]. In the present work, the WT *Arabidopsis* seedlings were treated with repeated wounding. Consistent with the previous reports, the repeated wounding caused the decrease in leaf area, fresh weight, dry weight, and root length (Fig. 1).

Theoretically, wounding would inevitably cause the release of ATP from the cytosol into the extracellular matrix, since ATP is omnipresent in cytoplasm and a breakage of plasma membrane by wounding provides a passive route of cytoplasm release [12, 33]. Sun et al. [43] observed an increase in the concentration of ATP in the extracellular fluid, which presented at wound sites of *Arabidopsis* leaves. Song et al. [12] observed that the exATP level in the roots of *Arabidopsis* seedlings reached a transient maximum at 15 min after wounding, but this peak was rapidly abolished at 30 min after wounding. We showed here that the seedlings wounded repeatedly had higher exATP than the un-wounded seedlings, and the repeated wounding led to continuous increase in the exATP level in the wounded seedlings (Fig. 2). Thus, it seems that late wounding can cause more release of exATP, compared to early wounding.

We also noted that after 21th day of the growth, the exATP levels in the *p2k1-3* mutant was higher than those in WT under either unwounded or wounded condition, although such difference was not always significant at the tested time points (supplement Table 1). Previous work by Deng et al. (2015) found that the ectopically expressed APYRASE, which is well known to decrease exATP level, has potential to enhance vesicular trafficking in *Arabidopsis*, which is also an important mechanism for plant exATP release [44]. This implies a possibility that decrease of exATP level or decrease in exATP perception could stimulate release of exATP by vesicular trafficking. Thus, we assume that the mutation in P2K1 could increase exATP secretion by enhancing vesicle trafficking, which exerts an unstable contribution to exATP level, since vesicle-mediated secretion is highly dynamic. Future studies are expected to reveal the mechanisms for the effects of P2K1 on exATP level.

Although the potential mechanism of this continuous increase of exATP level during the repeated wounding is complex and unclear, it has been proposed that exATP as a DAMP (damage-associated molecular pattern) signal could be a potential molecule required for the regulation of the defence responses of plants to pathogen infection or herbivore attack [13, 45]. However, whether plant exATP also plays a role in the wound-induced growth degradation is unclear. As induced above, the potential biological effects or functions of exATP can be revealed by exogenous application of ATP [7, 13]. For example, the elevation of cytosolic free calcium ([Ca^2+^]_cyt_) level is usually used as the indicator of the exATP-induced response [46]. Tanaka et al. [47] revealed that the concentrations of exogenous ATP as higher as 100 μM can trigger the elevation of [Ca^2+^]_cyt_ level in *Arabidopsis* seedlings. Otherwise, Stacey and his colleagues demonstrated that applied exogenous ATP at 200 μM or higher concentrations can induce immune response of *Arabidopsis* seedlings (extracellular ATP elicits DORN1-mediated RBOHD phosphorylation to regulate stomatal aperture; S-acylation of P2K1 mediates extracellular ATP-induced immune signaling in *Arabidopsis*). It is noted that exogenous ATP from 100 to 1,000 μM elevated [Ca^2+^]_cyt_ level of *Arabidopsis* seedlings in a dose-dependent manner, while the maximum binding capacity of the purified P2K1 to ATP was presented at 200 nM ATP by the saturation binding assays [21]. This is not surprising. In theory, when ATP was exogenously applied into plant tissue, only small proportion of ATP can bind the exATP receptors, since the natural components of the cuticle as the barriers can prevent polar compounds entering into the plants [48]. Thus, exogenous ATP at a concentration higher than physiological level is commonly employed to discover the possible physiological role of exATP. But, such application in excess of the physiological level of exATP could cause some artifactual effects on plants, and it is also difficult to determinate how much ATP in those applied exogenously can be actually perceived by the receptor. Hence, besides using exogenous ATP, the actual role of exATP in plants would be assessed by using exATP receptor mutant. In our experiments, it was shown that, similar to repeated wounding, repeated application of 0.1–5 mM exogenous ATP caused the decrease in leaf area, fresh weight, dry weight, and root length of *Arabidopsis* seedlings (Fig. 3), suggesting that exATP could have potential to inhibit plant growth. More importantly, the mutation in exATP receptor decreased the sensitivity of plant growth to the repeated wounding and exogenous ATP (Figs. 4, and 5, Table 1). Thus, when plants are subjected to the repeated wounding, the increase of exATP could play a role in inhibiting plant growth by binding its receptor, P2K1.

It is widely known that the JA content is enhanced upon tissue damage and JA is a critical regulator of the plant responses to wounding [35, 49]. We evaluated that function of JA in the wound-induced growth degradation. It was observed that IBU can partially rescue the wound-induced decrease of growth, indicating that the wound-induced growth degradation is associated with the JA biosynthesis. This observation is consistent with the previous reports that exogenous JA can suppress the plant growth and repeated wounding had less effect on the growth of the mutant that is unable to synthesize JA, compared to the wild type plants [31, 50]. We also noted that MYC mutation partially rescued the wound-induced decrease of leaf area and root length but had no significant effects on fresh and dry weight of the wounded seedlings. This could be not surprising, since JA can activated various downstream factors, including MYC2 /JASMONATE INSENSITIVE1 (JIN1), MYC3, MYC4, WD-repeat/bHLH/MYB complex, MYB21, MYB24, MYB57, and the IIId bHLH factors [51, 52]. These results suggest that the JA-signaling is involved in the regulation of the wound-induced growth degradation, and JA biosynthesis is vital for this process. MYC2 could be required, but is not sufficient, for the JA-mediated growth degradation.

As the results shown above, either the mutation in exATP receptor or application of IBU can partially rescued the wound-induced decreases of all of the growth parameters tested (Figs. 4 and 6). Thus, in the further work, IBU was applied exogenously to the *p2k1-3* mutation seedlings to evaluate the rescued degree of the wound-induced growth degradation by the combination of *p2k1-3* mutation and IBU, compared with either by *p2k1-3* mutation or IBU alone. The results showed that the combination of P2K1 mutation and IBU application did not produce additive rescue efficacy in attenuating the wound-induced growth degradation, compared with either the P2K1 mutation or IBU application alone (Fig. 7). In addition, under the repeated wounding, P2K1 mutation can partly rescue the wound-induced growth degradation, whereas this mutation had no significant impact on growth of the wounded seedlings that were repeatedly treated with IBU (Fig. 7). This indicate that the regulation of the wound-induced growth degradation by exATP could be linked to the JA signaling pathway.

In the last decades, the mode of action of JA has been well studied. The biosynthesis of JA is induced in plants exposed to biotic or abiotic stresses, including necrotrophic pathogen attacks, chewing insect herbivores, and wounding [36, 37, 39, 53]. Jasmonate ZIM-domain (JAZ) proteins is key negative regulator of JA signaling, which can block the activity of JA-specific master transcription factors [54–57]. When intracellular levels of JA rises above a threshold concentration, the induced JA binds to the JA receptor, coronatine-insensitive1 (COI1), and promotes the binding of JAZ proteins to COI1. As the result, JAZ proteins are degraded through an ubiquitination system, and this permits the various transcription factors to activate downstream target genes that are up-regulated by JA signaling [52, 58]. Interestingly, Tripathi et al. [58] found that exogenous addition of ATP decreased the JAZ stability, thus enhancing plant defense through reinforcing activation of JA signaling. Thus, it is reasonable to assume that the increase of exATP level by repeated wounding could decrease the JAZ stability by activating P2K1, thus being involved in the JA-regulated growth degradation by repeated wounding.

As described by the previous works, JA-signaling pathway activates defence responses when plants were threatened by necrotrophic pathogen attacks, chewing insect herbivores, and wounding, although the “side effect” of the wound-induced JA is to stunt plant growth [31, 35]. In fact, plants in natural environment have the ability to recognize and respond to threats and continuously integrate the information to tailor their growth, development and defensive capabilities in ways that optimize fitness [36, 52, 56]. In the last decades, tremendous progress has been made in understanding how plant exATP can also regulate the physiological process and gene expression related to defense responses [56–58]. As proposed by some researchers, exATP can serve as a signal of damage-associated molecular patterns (DAMPs) for the regulation of the defence responses of plants to pathogen infection and herbivore attack [36, 37, 39, 45, 53]. Otherwise, much works also revealed that exATP is integrated with other signaling pathways, such ROS, NO, and Ca^2+^ [10, 12, 17, 18, 59]. Our present work further indicates that exATP could be a regulator for the stunted growth of plants by repeated wounding. Hence, exATP could be a potential molecule regulating the balance between growth and defense in plants.

Benefit from the physiological function of exATP, exogenous ATP treatment has been employed in recent year as an innovative approach to ameliorating stress, retarding senescence and preserving quality of horticultural crops during postharvest storage [60–62]. And, as a small molecule, ATP has many advantages such as simple structure and easy preparation [63]. Further study in this area would lay a foundation for the actual application of exATP-based regulation agent in adjusting or optimizing the plant growth and defense.

## Methods

### Chemicals, growth condition and treatments

The seeds of *Arabidopsis thaliana* wild-type (WT, Columbia Col-0), *p2k1-3* mutant (Salk_042209, obtained from the Arabidopsis Biological Resource Center at Ohio State University, in which T-DNA disrupted the gene coding P2K1), and *myc2-2* mutant (Salk_083483) [64] were sown on pre-wetted media containing vermiculite and soil (1:2 in v/v). In order to avoid the possible influence of inconsistent growth conditions on the experimental results, the same type of *Arabidopsis* seedlings with the same treatment was placed on the same layer of culture rack, and the *Arabidopsis* seedlings on the separate layer of culture rack were arrayed with the same modality. After then, the plants were cultivated at 25℃ on a 16 h light: 8 h dark regime at 100 μmol photon m^−2^ s^−1^ PAR (photosynthetically active radiation).

The repeated wounding was performed as described by Zhang and Turner [31]. The first wounding was exerted when the *Arabidopsis* seedlings was 21 days old. For each occasion of wounding, one leaf of the seedling was wounded with forceps having serrated teeth. The same wound event was repeated on another leaf every one day and data were recorded every two day thereafter until d 31 of growth.

For the repeated chemical treatment, different concentration ATP-Na_2_ (0.1, 0.5, 1, 2.5, or 5 mM) and 20 μM IBU were prepared by dissolving the compounds with deionized distilled water and the PH value of the solutions was adjusted to 6.5. The first spray was applied when the *Arabidopsis* seedlings was 21 days old. For each occasion of spray application, the leaves were sprayed from multiple angles until the leaves were wet and solution ran off. The leaves of the seedling were sprayed with the solvent alone under the same conditions were used as the controls. The same event was repeated every one day and data were recorded every two day thereafter until d 31 of growth.

### Measurements of plant area, root length, fresh weight and dry weight

Images of the area of the all leaves of the seedlings were acquired using camera and were stored in JPG format. The area of leaves of the seedlings was calculated by Adobe Photoshop software with the help of the image of the known area. For the measurement of root length, the roots were rinsed with distilled water and the length of the tap root was measured with a ruler at an accuracy of mm. For the measurement of fresh and dry weight, the sample of the seedlings was blotted dry with filter- paper to remove any surface water, and was then immediately weighed to acquire fresh weight. The sample was dried in a drying box at 80 °C for 2 d, until a constant weight was obtained.

### Measurement of extracellular ATP

The leaves were submerged with deionized distilled water for 5 min for harvesting the exATP released, and then the liquid medium for the exATP measurement was placed on ice and the ATP content in a 100 μl of sample was assayed by using a commercial ATP assay kit (Beyotime, BioTechnology, China), which includes firefly luciferase and luciferin, based on the reaction ATP + luciferase + luciferin → oxyluciferin + AMP + CO_2_ + photon emission. The reaction is known to be strictly specific for ATP, i.e. other nucleotides are not suitable substrates and do not cause photon emission, and the production of photon emission from this reaction is proportional to the concentration of ATP [65]. The measurement was performed according to the instructions from the manufacturer and the light emission from the sample was measured with a GloMax Multi JR Luminometer (promega). The amounts of ATP were calculated from a standard curve by known concentrations of ATP. Same amount of the deionized distilled water that did not touched any plant tissue was handled identically, in which the ATP content was measured under the same conditions as the background. The measured content of exATP from the plant tissue were corrected by the subtraction of the ATP content from the background.

### Statistical analysis

Each value represents the mean ± standard deviation (SD) from at least four independent replicates. The data were statistically evaluated with t-test methods. All analyses were performed using SPSS software (version 13.0, SPSS).

## Supplementary Information

**Additional file 1: Table S1.** Effect of the repeated wounding on the leaf exATP level of the WT and *p2k1-3* mutant seedlings. The values represent means ± SD from twenty independent seedlings. The values in the control (21 days) were set to 1.000 to facilitate the comparison among the different treatments. The means denoted by the same letter did not significantly differ at *P* < 0.05 among the different treatment.

## Data Availability

The datasets used and/or analyzed during the current study are available from the corresponding author on reasonable request.
