# Supplementary material for: The involvement of extracellular ATP in regulating the stunted growth of Arabidopsis plants by repeated wounding
Source: BMC Plant Biol. 2022 Jun 8;22:279. doi: 10.1186/s12870-022-03656-z (PMC9175478; doi:10.1186/s12870-022-03656-z)
Supplement: Supplementary file 1 — Additional file 1: Table S1. Effect of the repeated wounding on the leaf exATP level of the WT and p2k1-3 mutant seedlings. The values represent means ± SD from twenty independent seedlings. The values in the control (21 days) were set to 1.000 to facilitate the comparison among the different treatments. The means denoted by the same letter did not significantly differ at P < 0.05 among the different treatment. [file 12870_2022_3656_MOESM1_ESM.docx]

The involvement of extracellular ATP in regulating the stunted growth of *Arabidopsis* plants by repeated wounding

Zhenzhen Shi; Hanqi Wang; Yuejing Zhang; Lingyun Jia; Hailong Pang; Hanqing Feng^a,^* Xin Wang

a College of Life Science, Northwest Normal University, 730070, Lanzhou, Gansu, China

1Corresponding author: fax: (+86) 0931-7971207; e-mail: fenghanq@nwnu.edu.cn

* Author for correspondence and reprint requests

| **exATP level/Day** | **21** | **23** | **25** | **27** | **29** | **31** |
| --- | --- | --- | --- | --- | --- | --- |
| **WT-control** | 1.000 ± 0.101^c^ | 1.027 ± 0.075^c^ | 1.132 ± 0.116^c^ | 1.171 ± 0.132^c^ | 1.215 ± 0.077^c^ | 1.294 ± 0.118^b^ |
| **WT-wounded** | 1.293 ± 0.071^b^ | 1.465 ± 0.030^ab^ | 1.583 ± 0.133^ab^ | 1.705 ± 0.268^b^ | 1.855 ± 0.158^b^ | 1.949 ± 0.215^ab^ |
| ***p2k1-3*-control** | 1.278 ± 0.075^b^ | 1.370 ± 0.148^bc^ | 1.421 ± 0.148^bc^ | 1.494 ± 0.199^bc^ | 1.530 ± 0.155^bc^ | 1.594 ± 0.074^b^ |
| ***p2k1-3*-wounded** | 1.578 ± 0.124^a^ | 1.838 ± 0.148^a^ | 1.954 ± 0.164^a^ | 2.183 ± 0.272^a^ | 2.498 ± 0.211^a^ | 2.635 ± 0.390^a^ |

**Table S1** Effect of the repeated wounding on the leaf exATP level of the WT and *p2k1-3* mutant seedlings. The values represent means ± SD from twenty independent seedlings. The values in the control (21 days) were set to 1.000 to facilitate the comparison among the different treatments. The means denoted by the same letter did not significantly differ at *P* < 0.05 among the different treatment.
